# MiR-155-5p positively regulates CCL17-induced colon cancer cell migration by targeting RhoA

**DOI:** 10.18632/oncotarget.14841

**Published:** 2017-01-27

**Authors:** Amr A. Al-Haidari, Ingvar Syk, Henrik Thorlacius

**Affiliations:** ^1^ Department of Clinical Sciences, Section of Surgery, Lund University, 20502 Malmö, Sweden

**Keywords:** chemokines, chemotaxis, metastasis, microRNA, colon cancer

## Abstract

Colorectal cancer is the second most common cause of cancer-related death, which is due to migration of tumor cells to distant sites of metastasis. Accumulating data indicate that mciroRNAs play an important role in several aspects of colon cancer cell biology. Herein, we examined the role of miR-155-5p in colon cancer cell migration induced by the CCL17-CCR4 axis in HT-29 colon cancer cells. We found that miR-155-5p knockdown in serum starved colon cancer cells decreased CCL17-induced cell chemotaxis. Moreover, knocking down miR-155-5p markedly decreased CCL17-provoked activation of RhoA in colon cancer cells. Bioinformatics analysis predicted two putative binding sites in the AU-rich element at the 3′-UTR of RhoA mRNA. MiR-155-5p binding to RhoA mRNA was verified using a target site blocker and functionally validated by RNA immunoprecipitation assays, showing that miR-155-5p-dependent regulation of RhoA mRNA is mediated by AU-rich elements present in the 3′-UTR region. Taken together, these results show that miR-155-5p positively regulates RhoA mRNA levels and translation as well as cell migration in serum starved colon cancer cells and indicate that targeting miR-155-5p might be a useful strategy to antagonize colon cancer metastasis.

## INTRODUCTION

Colorectal cancer is the third most prevalent cancer and the second most frequent cause of cancer-related death in the world [1, 2]. Metastasis is the dominant cause of death in patients with colon cancer [3]. The mechanisms behind colon cancer metastasis remains elusive but accumulating data indicate that increased expression of adhesion molecules and chemokines facilitate colon cancer migration and spread [4]. On top of cell migration, chemokines have been implicated in additional aspects of malignant transformation, such as proliferation, survival, and angiogenesis [5]. Convincing data have shown that colon cancer cells express several types of chemokine receptors. For example, it has recently been reported that colon cancer cells express CCR4, the receptor of CCL17, and that CCR4-CCL17 interactions mediate colon cancer cell migration [6]. Additional studies have demonstrated that the CCR4-CCL17 axis can promote breast cancer metastasis to the lung [7]. Cell migration is a dynamic and complex process involving multiple specific transcription factors, including small (~21 kDa) guanosine triphosphatases of the Ras-homologus (Rho) family, such as Rho A-C, Cdc42, and Rac1 [8]. Indeed, CCL17/CCR4-dependent colon cancer cell migration is associated with increased activity of RhoA and can be inhibited by targeting Rho-kinase function [6]. However, the detailed intracellular mechanisms regulating RhoA-dependent gene translation and chemotaxis are not known.

MicroRNAs (miRNAs) are short non-coding RNAs that function as post-transcriptional regulators of gene expression in effector complexes containing a core argonaute protein (AGO) [9, 10]. In general, miRNAs are predominately considered to cause downregulation of mRNA expression; however, under certain conditions miRNAs can upregulate expression of distinct mRNAs [11–13]. For example, serum starvation, which puts cells in a state of quiescence, has been shown to favor mRNA expression by certain miRNAs [13–15]. MiR-155 is overexpressed in different types of tumors, including colon cancer [16] and high expression of miR-155 correlates with poor prognosis in colorectal cancer patients [17] suggesting a pro-carcinogeneic role of miR-155. Increased RNA translation induced by miRNAs has been shown to be mediated via AU-rich elements (AREs) in the 3′ untranslated region (UTR) of mRNA for instance AUUA and AUUUA, as well as adjoining non-AU sequences [18] Notably, investigations have reported that miR-155 can regulate migration and invasion of certain tumor cell types, such as breast, pancreatic, hepatocellular and nasopharyngeal cancers [19–22]. However, the role and mechanism of MiR-155 in regulating chemokine-induced colon cancer cell migration is not known.

Based on the considerations above, we hypothesized that should be added after hypothesized miR-155-5p might regulate CCL17-induced and CCR4-dependent migration of colon cancer cells via RhoA function. For this purpose, we used a human colon cancer cell line transfected with AntagomiR-155-5p.

## RESULTS

### Downregulation of miR-155-5p inhibits migration of colon cancer cells

**Figure 1:**
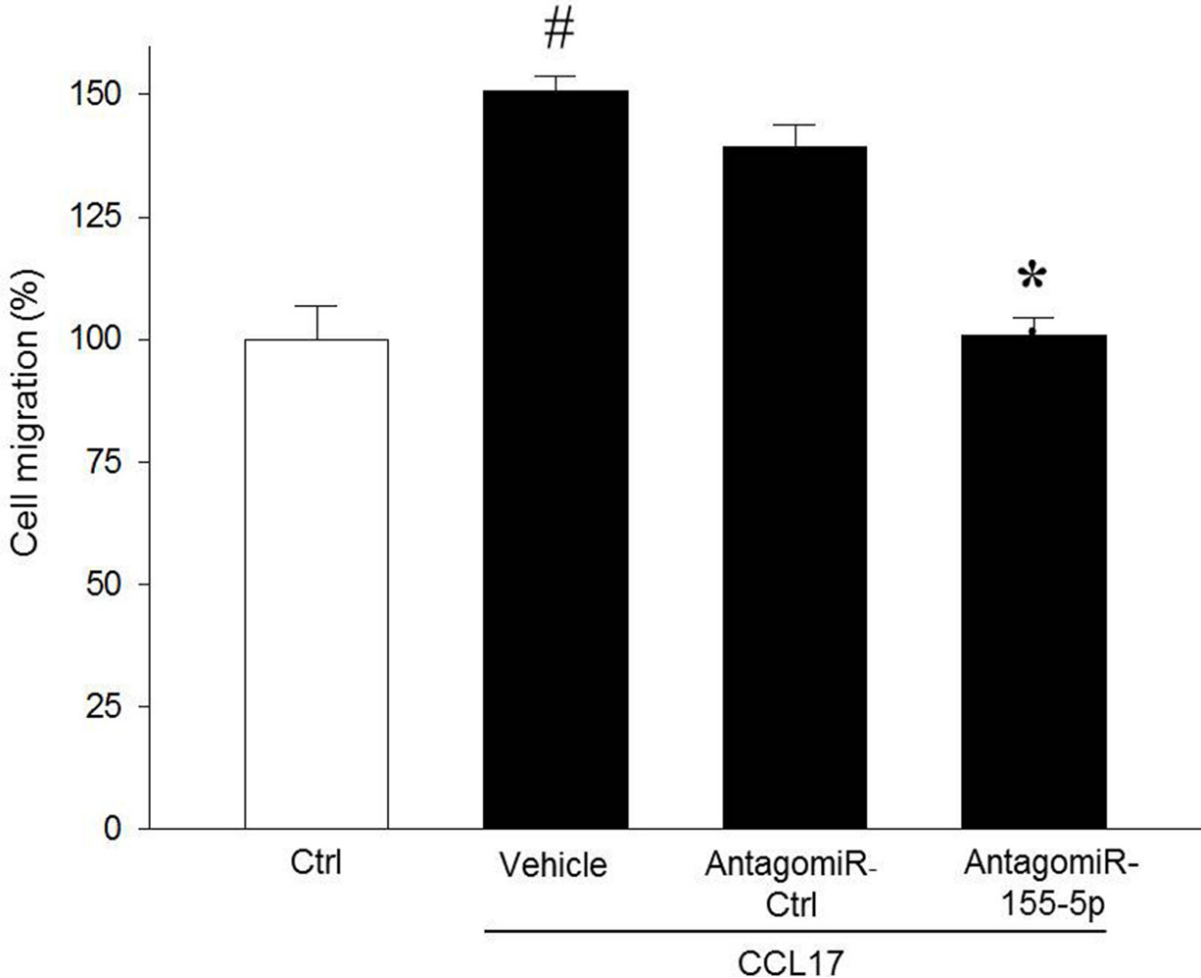
MiR-155-5p regulates colon cancer cell migration AntagomiR-155-5p (200 nM) decreased CCL17-induced HT-29 colon cancer cell migration. Cells were counted microscopically using 10 High Power Fields in five different fields. Migration index was calculated as the ratio of the number of migrated cells on wells containing CCL17 divided by the number of cells in the control wells. ^#^*P* < 0.05 versus negative ctrl and *P < 0.05 versus CCL17+ AntagomiR ctrl. All data are expressed as mean ± SEM and *n* = 4.

To analyze the function of miR-155-5p in colon cancer cells, miR-155-5p was knocked down in serum starved HT-29 colon adenocarcinoma cell line using AntagomiR-155-5p. Transfection with AntagomiR-155- 5p dose-dependently decreased miR-155-5p expression in HT-29 cells (Supplementary Figure 1A). For example, transfection with 200 nM of AntagomiR-155-5p reduced miR-155-5p expression by 77% (Supplementary Figure 1A). Importantly, AntagomiR-155-5p transfection had no effect on tumor cell viability (Supplementary Figure 1B). To investigate whether suppression of miR-155-5p affects colon cancer cell migration, transwell migration assays were performed with CCL17 as a chemoattractant. We found that transfection with AntagomiR-155-5p abolished CCL17-induced colon cancer cell migration compared with control (ctrl) or AntagomiR ctrl (Figure 1).

### miR-155-5p inhibition decreases RhoA activity

**Figure 2:**
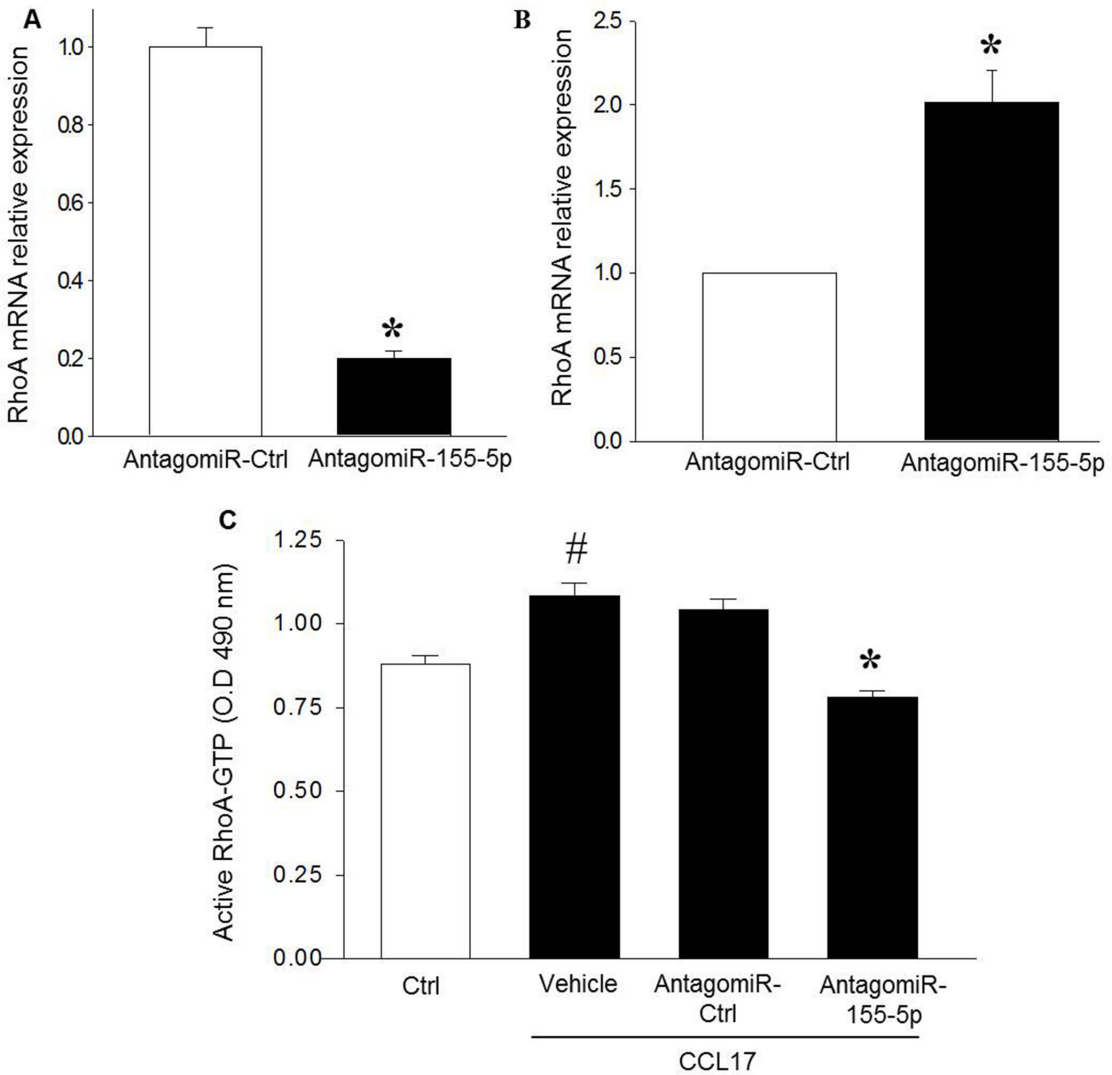
AntagomiR-155-5p reduces RhoA expression and activity in colon cancer cells (**A**) transfection by AntagomiR-155-5p (200 nM) downregulates RhoA mRNA expression in serum starved HT-29 colon cancer cells. Relative expression was demonstrated using qRT-PCR where beta actin was used as housekeeping gene and expression determined using 2^–ΔΔCT^ method. (**B**) QRT-PCR of RhoA mRNA expression in response to miR-155-5p knockdown in serum-grown HT-29 cells. Relative expression was normalized to beta actin and determined using 2^–ΔΔCT^ method. Data represents mean ± SEM and (*n* = 4). *P < 0.05 versus AntagomiR control. (**C**) AntagomiR-155-5p (200 nM) reduced CCL17-induced RhoA activation in serum starved HT-29 colon cancer cells. Data represent mean ± SEM and *n* = 4. ^#^*P* < 0.05 versus negative ctrl and *P < 0.05 versus AntagomiR ctrl.

Knowing that CCL17-induced colon cancer cell migration is dependent on RhoA signaling [6], we next asked whether miR-155-5p might regulate CCL17-evoked activation of RhoA. It was found that AntagomiR-155-5p transfection markedly decreased RhoA mRNA expression in HT-29 starved cells (Figure 2A) while knocking down miR-155-5p in normal serum conditions increased RhoA mRNA (Figure 2B and Supplementary Figure 1C). Co-incubation with CCL17 significantly increased RhoA activity in HT-29 cells (Figure 2C). Moreover, we observed that knocking down miR-155-5p also abolished CCL17-induced activation of RhoA (Figure 2C).

### Target prediction of miR-155-5p

**Figure 3:**
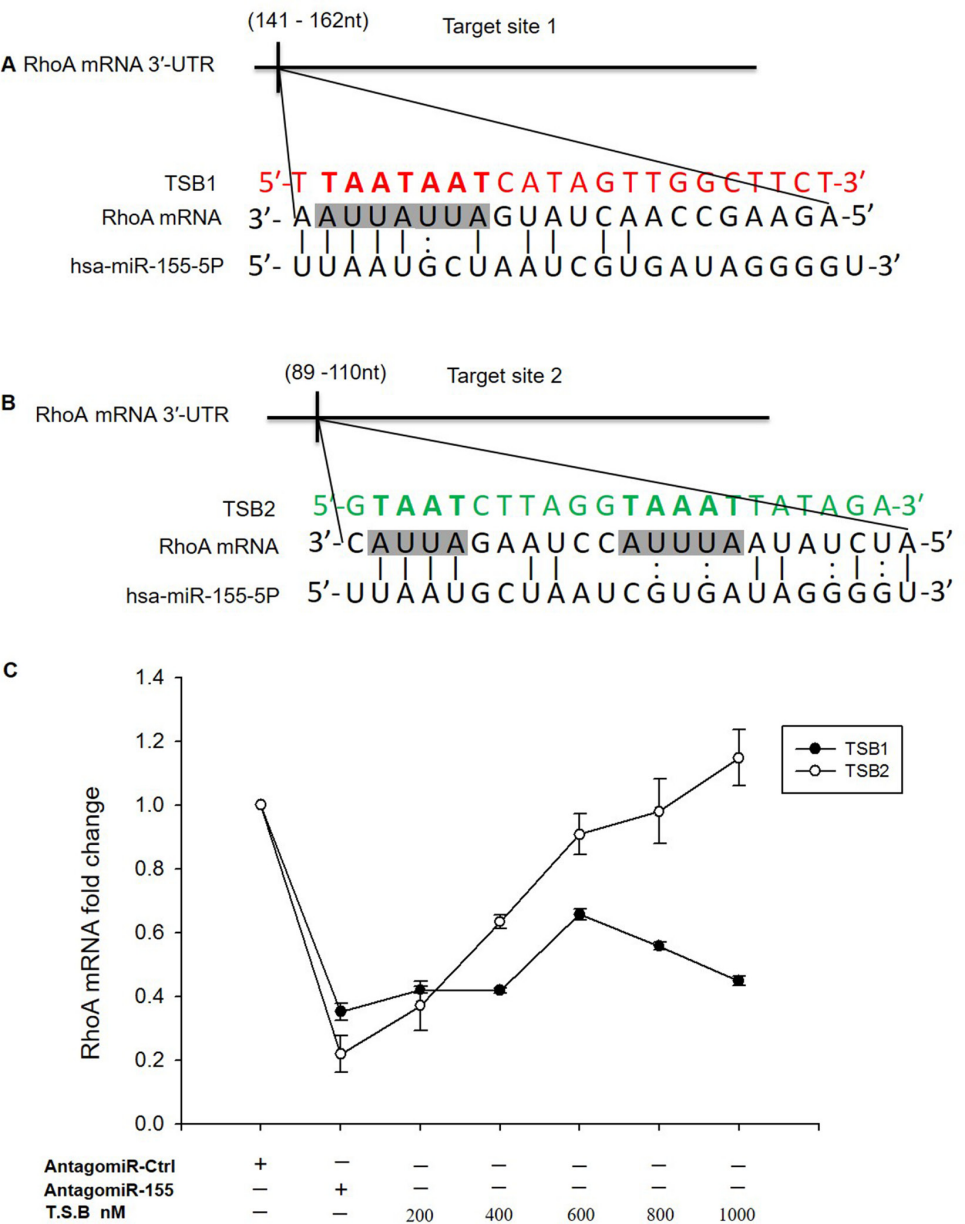
RhoA is a direct target of miR-155-5p (**A**) predicted target site 1 of miR-155-5p in RhoA mRNA 3′-UTR sequence containing an ARE (AUUA) motif in shaded box. The seeding region of miR-155-5p complementary to AUUA was blocked using TSB1, red sequence. (**B**) target site 2 of miR-155-5p in the RhoA mRNA 3′-UTR sequence is depicted in shaded boxes containing two AREs motifs, AUUA and AUUUA, blocked by TSB2, green sequence. (**C**) TSB2 dose-dependently reversed the effect of AntagomiR-155-5p on RhoA mRNA expression in serum starved HT-29 colon cancer cells. Data represent mean ± SEM and *n* = 3.

We asked if RhoA mRNA could be a direct target for miR-155-5p in HT-29 cells and whether the positive regulation profile on cell migration is related to direct binding of miR-155-5p to RhoA mRNA. RNAhybrid target prediction analysis revealed five different target hits in the 3′-UTR of RhoA mRNA containing miR-155-5p recognition sites (Supplementary Figure 2). However, based on potentially existing evidences on the role of ARE sites in RNA translation, [18] we limited our analysis to the regulatory ARE sites in the 3′-UTR of RhoA mRNA. Strikingly, we found two potential sites, i.e., target site 1 containing the tetramer AUUA which is complementary to the 4′-mer of the miR-155-5p seeding region and target site 2 containing the tetramer AUUA and the pentamer AUUUA (Figure 3A and 3B). The function of these sites was examined by use of TSBs. Again, it was found that transfection with AntagomiR-155-5p markedly reduced RhoA mRNA expression in HT-29 (Figure 3C). Interestingly, we observed that co-transfection with TSB1 which blocks target site 1 was unable to reverse the effect of AntagomiR-155-5p (Figure 3C). In contrast, co-transfection with TSB2, which blocks target site 2, dose-dependently increased expression of RhoA mRNA in HT-29 carcinoma cells transfected with AntagomiR-155-5p (Figure 3C), suggesting that miR-155-5p interacts with this specific target site in RhoA mRNA.

### RhoA mRNA is a direct target of miR-155-5p

**Figure 4:**
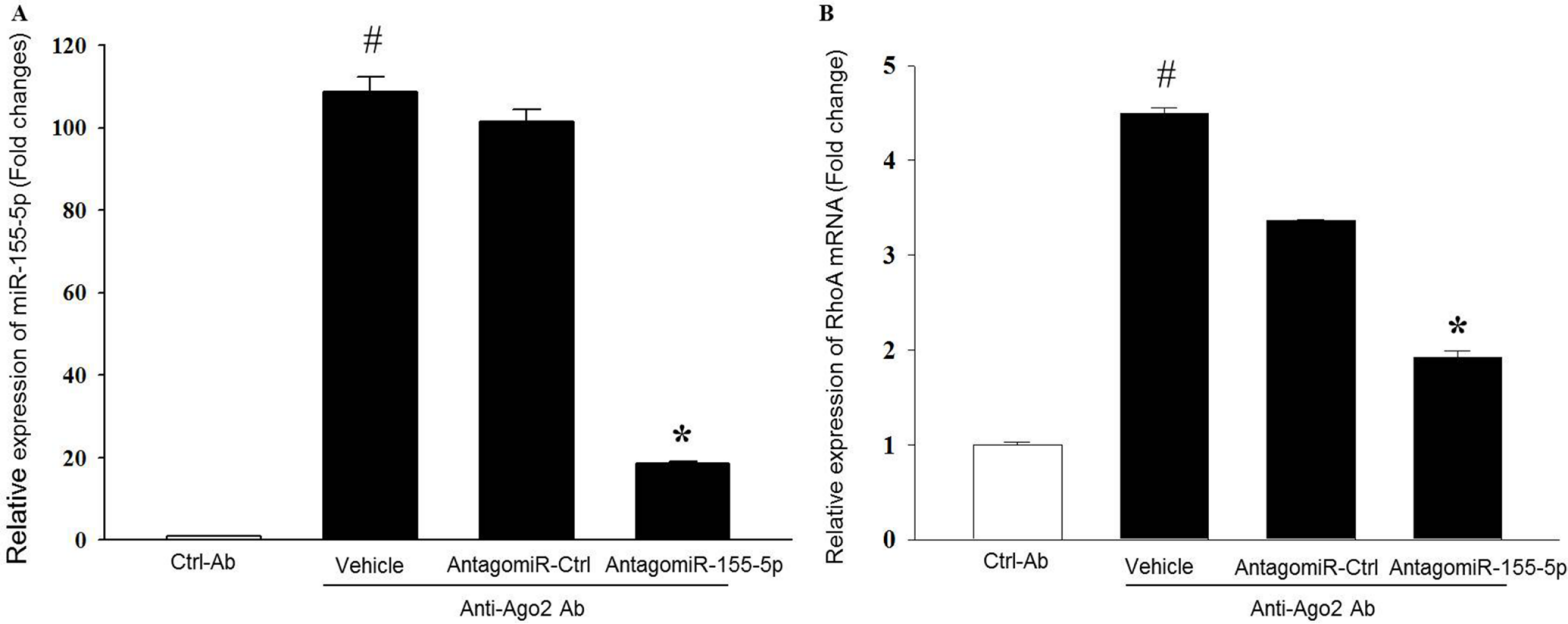
MiR-155-5p is associated with RhoA mRNA in Ago2 immunoprecipitates The amount of miR-155-5p and RhoA mRNA was measured in input RNA used for the RIP assays by qRT-PCR. AntagomiR-155-5p decreased relative enrichment of (**A**) miR-155-5p and (**B**) RhoA mRNA in Ago2 immunoprecipitates. Data are expressed as mean ± SEM and *n* = 4. Data are expressed as fold change compared to anti-IgG ctrl. U1 snRNA and beta actin were used as housekeeping genes for miR-155-5p and RhoA mRNA, respectively. ^#^*P* < 0.05 versus ctrl-Ab and *P < 0.05 versus anti-Ago2-AntagomiR ctrl treated cells.

To verify the association between miR-155-5p and RhoA mRNA in HT-29 cells, we used RIP with anti-Ago-2 beads. The Ago2 protein is the core and catalytic component of RISC [24]. Since microRNAs are known to be present in the cytoplasm as miRNA-ribonucleoprotein complex (miRNPs) and their functions are mediated by Ago2 protein [25], RIP assays were performed on AntagomiR-155-5p transfected HT-29 cells to determine the association between miR-155-5p and RhoA mRNA. Levels of mRNA levels in the immunoprecipitates were detected by use of qRT-PCR. We observed that miR-155-5p was preferentially enriched (109-folds) in Ago2-containing miRNPs relative to ctrl-IgG immunoprecipitates (Figure 4A). In contrast, enrichment of miR-155-5p was reduced by 81% in AntagomiR-155-5p transfected HT-29 carcinoma cells (Figure 4A). Moreover, the anti-Ago2 antibody specifically pulled down nearly 5-fold more RhoA mRNA as compared to ctrl-IgG. Knocking down miR-155-5p reduced Ago2 antibody-induced enrichment of RhoA mRNA by 58% compared to AntagomiR Ctrl (Figure 4B).

## DISCUSSION

Tumor cell migration is a precondition for subsequent metastasis to distant organs [26]. In the present study, we examined posttranscriptional regulation of chemokine-induced colon cancer cell migration. We found that inhibition of miR-155-5p decreased RhoA activity and migration of HT-29 carcinoma cells in response to CCL17 stimulation. Moreover, we identified a functional target site of miR-155-5p at the 3′-UTR of RhoA mRNA in HT-29 cells. To our knowledge, this is the first report showing that miRNAs can directly regulate chemokine-induced colon cancer cell migration and suggest that targeting miR-155 might be a useful strategy to ameliorate the spread of colon cancer cells to distant sites.

MiRNAs have emerged as an important class of short noncoding RNAs regulating gene expression as well as an array of cellular functions, such as differentiation, growth, proliferation, adhesion, migration and apoptosis [27, 28]. MiR-155 has been reported to be overexpressed in colon cancer and previous studies have shown that RhoA is a direct target of miR-155 in different cell types, including breast cancer, endothelial, and epithelial cells [17, 29–34]. Considering the key role of RhoA in cancer cell migration [6, 35], it was of great interest to examine the role of miR-155 in CCL17-induced colon cancer cell migration in the present study. We found that AntagomiR-mediated knock down of miR-155-5p decreased CCL17-dependent cell migration without affecting colon cancer cell viability, suggesting that miR-155-5p is an important regulator of chemokine-induced directed colon cancer cell chemotaxis. This finding extends on a previous study showing that overexpression of miR-155 in HT-29 cells causes increased random migration [31]. Indeed, this adds colon cancer cells to the line of tumor cells, including hepatocellular, squamous, renal, pancreatic, and breast cancer cells, which exhibits miR-155-dependent migration [19, 36–38]. In this context, it is interesting to note that increased expression of miR-155 is associated with higher frequency of distant metastases in patients with colorectal cancer [31]. Thus, it could be speculated that miR-155-dependent tumor cell migration might at least be one mechanism explaining the increased risk of metastasis in patients with high expression of miR-155. Moreover, we observed that AntagomiR-induced knock down of miR-155-5p also attenuated RhoA mRNA expression and activity, suggesting that miR-155-5p is a positive regulator of RhoA in serum starved colon cancer cells. In general, miRNAs are considered to inhibit gene translation; however, accumulating data demonstrate that several miRNAs can cause increased RNA translation in cells under quiescence-like conditions, such as high cell confluence or serum starvation [13, 15]. Besides cellular conditions, different cell types respond differently to specific miRNAs. For example, miR-21 has been demonstrated to upregulate Bcl-2 in pancreatic carcinoma cells but downregulates Bcl-2 expression in breast and glioblastoma cancer cells [39]. Such context-dependent translation of some miRNAs targets is supported by our findings showing that knocking down miR-155-5p in non-serum starved colon cancer cells triggered increased expression of RhoA mRNA (Figure 2B and Supplementary Figure 1C). Considered together, these findings suggest that miR-155-5p constitutes a pro-carcinogenic miRNA in serum starved colon cancer cells via promotion of RhoA signaling and migration in response to CCL17 stimulation. To our knowledge, this study is the first report to show that miR-155-5p regulates colon cancer cell migration in response to chemokine signaling.

It is widely held that RhoA acts as a pro-oncogene and is often overexpressed in different kinds of tumors, including colon cancer [40]. RhoA plays an important function as a molecular switch in transducing extracellular signals to actin and microtubule cytoskeleton as an integrated part of cell migration [41]. As described above, we found that miR-155-5p is a positive regulator of RhoA mRNA expression and function in serum starved colon cancer cells. Positive regulation of mRNA translation by miRNAs is related to 3′-UTR target sites of miRNAs during cell cycle arrest [13–15]. In order to determine whether RhoA is a direct target of miR-155-5p, we performed bioinformatics analysis and found five target sites using RNAhybrid in the 3′-UTR of RhoA mRNA, showing that RhoA mRNA has multiple sites for miR-155 as described before [30, 32, 33]. However, our work was focused on ARE motifs specifically (AUUA and AUUUA) in the RhoA mRNA sequence considering that published data have shown that AREs present in the 3′-UTR of mRNAs could play a central role in activating RNA translation [12, 13, 18, 42]. We identified one region that was complementary to the seeding region of miR-155-5p with 4′-mer perfect binding (TS1) and another region that had both perfect at the seeding region and imperfect binding at its centered site (TS2). Previous reports indicated that binding to imperfect centered sites is as important as binding to seeding sites in augmenting miRNA and mRNA target interactions through imperfect binding [43]. We then designed specific blockers targeting these ARE sites at 3′-UTR of RhoA mRNA. Interestingly, we found co-incubation with one specific blocker targeting the TS2 ARE motifs (AUUA and AUUUA), which binds miR-155-5p at the seeding region and the centered site, dose-dependently reversed AntagomiR-155-5p-induced inhibition of RhoA mRNA expression, suggesting that this specific ARE region of 3′-UTR of RhoA mRNA is a functional target of miR-155-5p in serum starved colon cancer cells. Thus, this study identifies a novel target site regulating translational activation of RhoA mRNA by miR-155-5p. These results are in line with our findings showing that serum starved HT-29 colon cancer cells exhibited enrichment of RhoA mRNA and miR-155-5p levels in the Ago2 protein. It is important to note that miRNAs might act on multiple gene targets. In this context, it has been shown that GW182, which is a key protein in miRNA-mediated translation repression and part of the RISC complex, promotes miRNA induced translation in G0 state [44], and whether miR-155-5p could negatively regulate GW182 and thereby initiate translation activation remains to be elucidated. Increased translation can also be mediated by AREs-binding proteins, such as human antigen R (Hur), cytotoxic granule-associated RNA binding protein, and Tristetraprolin via direct or indirect miRNA binding [45–47]. For example, it has been reported that miR-155 increase tumor necrosis factor alpha mRNA stability and transcription via Hur in activated macrophages [48]. Knowing that the Hur protein is overexpressed in colon cancer cells it might be of value to study the role of Hur in mediating miR-155-induced translation of RhoA mRNA in serum starved colon cancer cells in future studies to improve the understanding of mechanisms regulating colon cancer cell migration and metastasis.

In summary, our data show that miR-155-5p positively regulates CCL17-induced RhoA activity and migration of serum starved colon cancer cells. Moreover, this effect of miR-155-5p was found to be mediated by specific ARE elements, AUUA and AUUUA, present in the 3′-UTR region of RhoA mRNA. These data do not only elucidate novel mechanisms regulating chemokine-dependent migration of colon cancer cells but might also help to develop more specific and effective strategies against colon cancer cell metastasis.

## MATERIALS AND METHODS

### Cells and reagents

The human epithelial colon adenocarcinoma cell line HT-29 was obtained from American Type Culture Collection (HTB-38, ATCC, Manassas, VA, USA). Cells were cultured in Dulbecco's Modified Eagle Medium (DMEM); (Sigma-Aldrich, Stockholm, Sweden), supplemented with 10% FBS, 100 U/ml penicillin, 100 μg/ml streptomycin at 37°C and 5% CO_2_. AntagomiR ctrl and AntagomiR-155-5p inhibitor (Life Technologies, Carlsbad, CA, USA) were used to evaluate the role of miR-155-5p by use of lipofectamine RNAimax transfection reagent (Life Technologies, Carlsbad, CA, USA). Chemokine CCL17 was purchased from Peprotech (Rocky Hill, NJ, USA). Anti-Ago2 clone 1B1-E2H5 RIP-certified and ctrl-IgG were purchased from (MBL international, Woburn, MA, USA). Target site blockers (TSB) LNA oligonucleotides were from Exiqon A/S (Vedbaek, Denmark).

### Cell transfection

HT-29 colon cancer cells at 60% confluency were starved overnight and on the next day 1 × 10^6^ cells were plated in a 6-well culture plate. Cells were reverse transfected with AntagomiR-155-5p (100 nM and 200 nM) or AntagomiR ctrl for 24 h using Lipofectamine RNAiMax (Life Technologies, Carlsbad, CA, USA) in Opti-MEM reduced serum media according to manufacturer's instructions. After 24 h, cells were harvested and expression of miR-155-5p and RhoA mRNA was analyzed by qRT-PCR. Briefly, RNA samples were extracted using Direct-zol RNA extraction kit (Zymo Research, Irvine, CA, USA) kit according to manufacturer's recommendations. 100 ng total RNA was used in each reaction and cDNA was synthesized using Mir-X™ miRNA First-Strand Synthesis Kit and miR-155-5p and RhoA mRNA were quantified using mir-X™ miRNA qRT-PCR SYBR^®^ kit (Clontech, Mountain View, CA, USA). The PCR primers used were as follows; hsa-miR-155-5p specific sense 5′- GGGTTAATGCTAATCGTGATAGGGGT-3′, RhoA sense; 5′-AGAGGTGTATGTGCCCACAGTGTT-3′, antisense; 5′-AGGCGATCATAATCTTCCTGCCCA-3′, U6 snRNA sense; GCTTCGGCAGCACATATACTA, antisense; CGA ATTTGCGTGTCATCCTTG, Beta actin sense; 5′-AGAG CCTCGCCTTTGCCGATCC-3′, antisense; 5′-CACATG CCGGAGCCGTTGTCG-3′. Expression of RhoA relative to beta actin, and miR-155-5p relative to U6 snRNA were determined using 2^- ∆∆^^CT^ method.

### Trypan blue dye exclusion assay

Viability of HT-29 colon cancer cells was quantitatively measured using trypan blue exclusion assay. After transfection by AntagomiR-155-5p (200 nM) and AntagomiR Ctrl at 24 h, cell suspensions were mixed with 0.4% Trypan blue stain. Total cells and viable cells (cells that excluded the blue stain) were counted using hemocytometer under a light microscope. All assays were performed in quadruplicate.

### Chemotaxis assay

Chemotactic response of HT-29 cells was evaluated by using 24-well cell migration chambers with 8 μm pore size inserts (Corning Coster, Corning, NY, USA). The Colon cancer cells were serum starved overnight and resuspended in Opti-MEM serum reduced media. The Cells were then transfected with either AntagomiR-155-5p (200 nM) or AntagomiR ctrl (200 nM) for 24 h. 1 × 10^6^ cells/ml were loaded in the inserts. DMEM with or without 100 ng/ml of CCL17 was added to the lower chambers and incubated for 3 h at 37°C (5% CO_2_). Non-migrated cells were removed by cotton swabs from the upper surface of the insert and cells on the lower surface of the insert membrane were fixed in ice-cold 100% methanol and stained with 0.5% crystal violet. All migrated cells were counted microscopically in at least 5 different fields. Migration index was then calculated as the ratio of the number of migrated cells divided by the number of cells in the control wells.

### RhoA activation assay

RhoA-GTP activity was measured using the G-LISA RhoA activation assay Biochem kit (Cytoskeleton Inc., Denver, CO, USA) according to manufacturer's instructions. Briefly, cells were serum starved overnight and reverse transfected by either AntagomiR-155-5p (200 nM) or AntagomiR-155 ctrl for 24 h. The next day, cells were stimulated with 100 ng/ml of CCL17 for 3 h and cells were trypsinized, washed by ice cold PBS and lysed in 0.35 ml lysis buffer of the kit on ice for 10 min. Then, cells were homogenized using a 20-gauge needle for 20 strokes on ice and centrifuged at 10 000 rpm for 20 min at 4°C. Supernatants were collected, snap frozen in liquid nitrogen and stored at -80°C until used. 50 μL of the supernatants were used for protein determination using Precision Red Advanced Protein Assay supplied with the kit (Cytoskeleton Inc., Denver, CO, USA). 1 mg/ml of protein was used for quantitative detection of active RhoA according to the manufacturer's recommendations. Absorbance was read at 490 nm using a microplate ELISA reader.

### Target site prediction and target site blockers (TSB) of miR-155-5p

RNAhybrid web-based bioinformatics target prediction algorithm was used to predict binding sites for miR-155-5p at the 3′-UTR of RhoA mRNA (http://bibiserv.techfak.uni-bielefeld.de/rnahybrid). However, we took into consideration the presence of regulatory ARE sites in RhoA mRNA specifically AUUUA and AUUA motifs and therefore our analysis was limited to ARE sites. To assess the function of the binding sites, we designed target site blockers, TSB1 and TSB2, (22 nucleotides) to bind selectively to sequences overlapping with the miR-155-5p ARE sites in the 3′-UTR of RhoA mRNA. The blockers were synthesized as fully phosphorothiolated Locked Nucleic acids in the DNA sequences to increase their affinity and selectivity for the target. Under serum starved conditions, the target site blockers TSB1_RhoA_miR-155-5p; 5′-TTAATAATCATAGTTGGCTTCT-3′ and TSB2_RhoA_miR-155-5p; 5′-GTAATCTTAGGTAAATT ATAGA-3′ were co-transfected with the AntagomiR-155-5p in different concentrations (200 – 1000 nM) in HT-29 colon cancer cells. RhoA mRNA levels were quantified using qRT-PCR. The potential target was functionally validated using RNA immunoprecipitation (RIP) assays.

### RIP assay

RIP has recently been utilized to identify mRNAs that are associated with the RNA-silencing machinery and therefore being targets of cellular miRNAs [23]. RIP was performed using the EZ-Magna RIP kit (Millipore, Billerica, MA, USA) following the manufacturer's protocol. Cells were scraped off 24 h after transfection with either AntagomiR-155-5p (200 nM) or AntagomiR ctrl. Cells were then lysed in complete RIP lysis buffer containing protease inhibitor cocktail, after which 100 μl of whole cell extract was incubated with RIP buffer containing magnetic beads conjugated with an anti-Ago2 antibody or ctrl-IgG antibody and rotated for 3 h at 4°C. After several washes samples were incubated with Proteinase K with shaking to digest proteins at 55°C. RNA was then isolated and concentrations were measured using a NanoDrop (ND-1000, Spectrophotometer, Thermo Scientific, Waltham, MA, USA). The co-immunoprecipitated (co-IP) RNA, including microRNA:mRNA complexes, were analysed by qRT-PCR to measure relative enrichment of miR-155-5p and RhoA mRNA.

### Statistical analysis

All statistical analyses were performed using SigmaPlot^®^ 10 software. For multiple comparisons Kruskal–Wallis One Way Analysis of variance on ranks followed by the Dunnett's post hoc test was used. *P-value* < 0.05 was considered significant.

## SUPPLEMENTARY MATERIALS FIGURES AND TABLES


