# Supplementary material for: MiR-155-5p positively regulates CCL17-induced colon cancer cell migration by targeting RhoA
Source: Oncotarget. 2017 Jan 27;8(9):14887–96. doi: 10.18632/oncotarget.14841 (PMC5362452; doi:10.18632/oncotarget.14841)
Supplement: Supplementary file 1 [file oncotarget-08-14887-s001.pdf]

MiR-155-5p positively regulates CCL17-induced colon cancer cell migration by targeting RhoA

Supplementary Materials

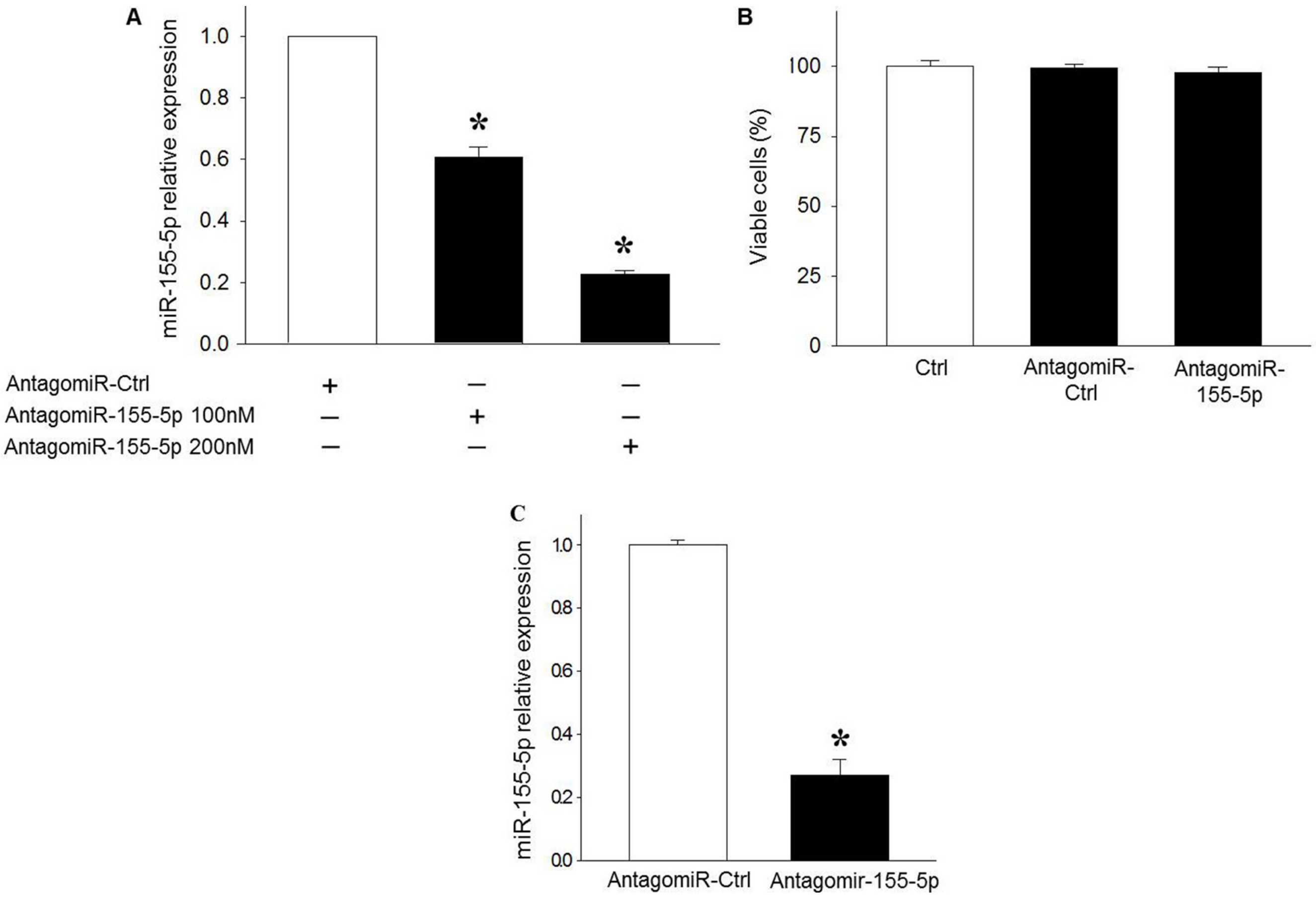

Supplementary Figure 1: Effect of AntagomiR-155-5p on RhoA and colon cancer cell viability. (**A**) Transfection by AntagomiR-155-5p for 24 h dose-dependently inhibits miR-155-5p expression in serum starved HT-29 colon cancer cells. QRT-PCR data were normalized against U6 snRNA housekeeping gene. *$P < 0.05$ versus AntagomiR ctrl. (**B**) viability of HT-29 colon cancer cells transfected with AntagomiR-155-5p (200 nM) or AntagomiR ctrl. All assays were performed in quadruplicate. (**C**) HT-29 colon cancer cells were grown in 10% serum and miR-155-5p was knocked down using AntagomiR-155-5p (200 nM) or AntagomiR control (200 nM) and relative expression was demonstrated using qRT-PCR and U6 snRNA used as housekeeping gene.

Version: RNAhybrid 2.2

searching
dataset: 1
mde of hsa-miR-155-5p: -45.499996
Individual hits

---

dataset: 1
Target: *RhoA*
length: 1063
MiRNA: *hsa-miR-155-5p*
length: 23

mfe: -23.0 kcal/mol
p-value: 1.000000e+00

Position: 18

```
target 5' A                AUU    A 3'
           GCCCUUAU GCGGUUA   UUGA
           UGGGGAUA UGCUAAU   AAUU
miRNA  3'          G       CGU      5'
```

plot as png,*jpeg* *or* ps *(in a new window)*

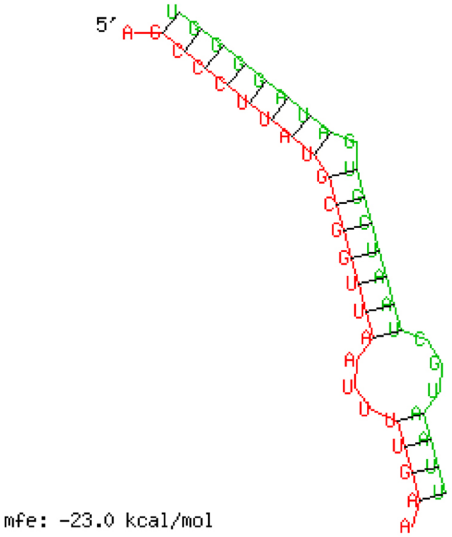

---

dataset: 1
Target: *RhoA*
length: 1063
MiRNA: *hsa-miR-155-5p*
length: 23

mfe: -22.7 kcal/mol
p-value: 1.000000e+00

Position: 951

```
target 5' A   A  AGUUC     GG     C    3'
           GCC CC     UCAUG  UGGCA
           UGG GG     AGUGC  AUCGU
miRNA  3'        AU        UA     AAUU 5'
```

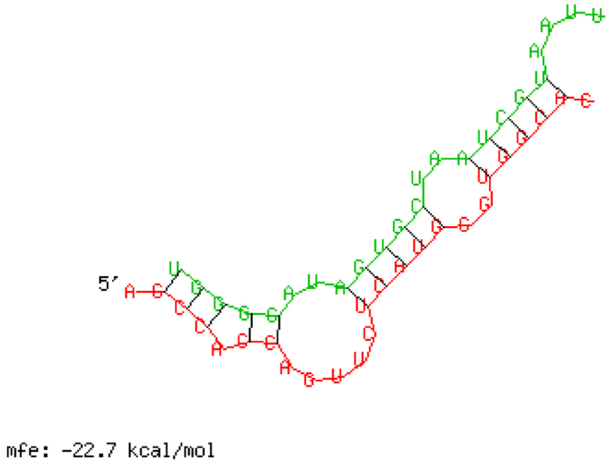

plot as png,*jpeg* *or* ps *(in a new window)*

---

dataset: 1
Target: *RhoA*
length: 1063
MiRNA: *hsa-miR-155-5p*
length: 23

mfe: -21.9 kcal/mol
p-value: 1.000000e+00

Position: 474

```
target 5' A    AAGU     CAGCUG     G    3'
           GCCC    UCAUG      UGGCA
           UGGG    AGUGC      AUCGU
miRNA  3'      GAU      UA         AAUU 5'
```

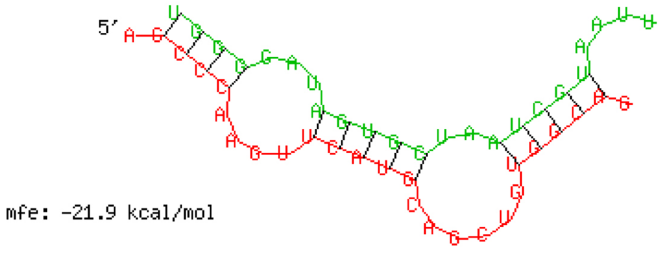

plot as png,*jpeg* *or* ps *(in a new window)*

---

dataset: 1
Target: *RhoA*
length: 1063
MiRNA: *hsa-miR-155-5p*
length: 23

mfe: -20.5 kcal/mol
p-value: 1.000000e+00

Position: 589

```
target 5' U     AGACUA   G       U   3'
            ACCCC      GAU UAGUAUU
            UGGGG      CUA AUCGUAA
miRNA  3'       AUAGUG           UU 5'
```

plot as png, jpeg or ps (in a new window)

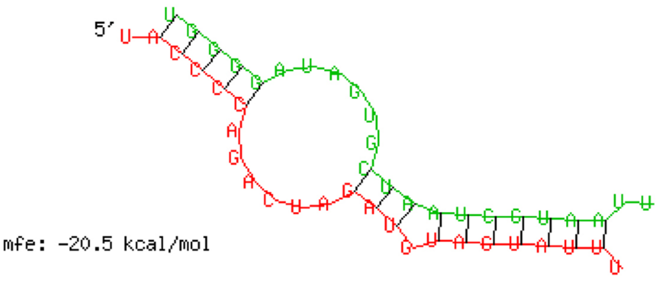

---

dataset: 1
Target: *RhoA*
length: 1063
MiRNA: *hsa-miR-155-5p*
length: 23

mfe: -19.9 kcal/mol
p-value: 1.000000e+00

Position: 791

```
target 5' U     A  GAG UG  CUA    A    3'
            ACCCC UG   C  GG   AGUA
            UGGGG AU   G  CU   UCGU
miRNA  3'          A   UG  AA      AAUU 5'
```

plot as png, jpeg or ps (in a new window)

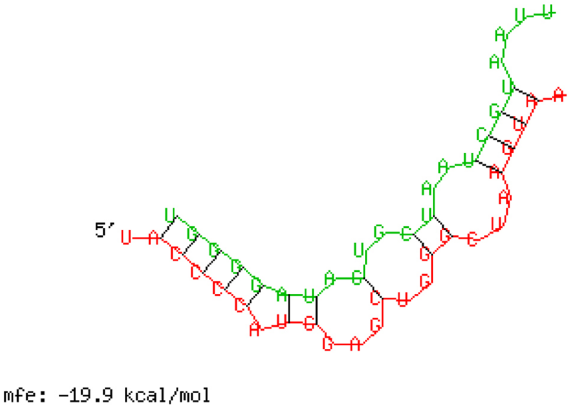

Supplementary Figure 2: RhoA is a direct target of miR-155-5p. Bioinformatics analysis of Predicted sites of miR-155-5p in 3′UTR of RhoA mRNA. The base–pairing (green: microRNA sequence; red: RhoA mRNA sequence) and the minimum free energy (mfe) of the binding of miR-155-5p and its targeting sequences were predicted using RNAhybrid program.
